# Human leukocyte antigen type does not improve risk stratification for SUNTRAC score in solid organ transplant recipients: A cohort study

**DOI:** 10.1016/j.jdin.2024.07.026

**Published:** 2024-08-31

**Authors:** Dominique Mosley, Christopher Madden, Jacqueline Ike, Isabelle T. Smith, Sarah Grossarth, Lee Wheless

**Affiliations:** a Vanderbilt University School of Medicine, Nashville, Tennessee; b State University of New York Downstate College of Medicine, Brooklyn, New York; c Meharry Medical College, Nashville, Tennessee; d Vanderbilt University College of Arts and Sciences, Nashville, Tennessee; e Quillen College of Medicine, East Tennessee State University, Johnson City, Tennessee; f Tennessee Valley Healthcare System VA Medical Center, Nashville, Tennessee; g Department of Medicine, Division of Epidemiology and Department of Dermatology, Vanderbilt University Medical Center, Nashville, Tennessee

**Keywords:** cohort study, epidemiology, organ transplant, skin cancer

*To the Editor:* The Skin and Ultraviolet Neoplasia Transplant Risk Assessment Calculator (SUNTRAC) scoring system aids in clinical decision-making for screening solid organ transplant recipients (SOTR) for cutaneous squamous cell carcinoma (cSCC).^1^ This system has yet to be evaluated with additional baseline clinical variables such as human leukocyte antigen (HLA).^2^^,^^3^ Our study investigates SUNTRAC’s performance with diverse skin cancers and HLA types.

Following Vanderbilt University Medical Center’s (VUMC’s) institutional review board approval VR54787, we collected data from VUMC’s research database and biobank on white SOTR. We previously described the cohort, which included all SOTR with transplant prior to 2020.^4^ Analyses were conducted between 10/2023 and 1/2024 on data ending 10/1/2023, ensuring all patients had at least 3 years of follow-up after transplant. Patients missing SUNTRAC variables or skin cancer dates were excluded. The primary outcome was time to histologically-verified skin cancers. When only notes were available that did not provide skin cancer type, it was listed as unknown. The primary exposure was HLA type. HLA types associated univariately with skin cancer were pooled into a single “high risk HLA” variable (Table I). We constructed risk stratification models for SUNTRAC or SUNTRAC plus HLA type in R v4.2.2 using the pec, cmprsk, and riskRegression packages. Model performance was assessed at 2 years since transplant using bootstrap C-statistic values derived from 200 cycles with replacement.

**Table I:** Characteristics of cohort

Mean age at transplant (SD, median)	51.6 (12.4, 53.8)
Gender, *n* (%)	
M	939 (62.6%)
F	560 (37.4%)
Transplanted organ type, *n* (%)	
Heart	233 (15.5%)
Lung	129 (8.6%)
Kidney	633 (42.2%)
Liver	504 (33.6%)
SUNTRAC group, *n* (%)	
Low risk	0
Medium risk	847 (56.5%)
High risk	636 (42.4%)
Very high risk	16 (1.1%)
Skin cancer, *n* (%)	
Any skin cancer	197 (13.1%)
cSCC	109 (7.3%)
BCC	71 (4.7%)
Melanoma	10 (0.7%)
Other and unknown	44 (2.9%)
HLA	
Low risk	752 (50.2%)
High risk∗	747 (49.8%)

*BCC*, Basal cell carcinoma; *cSCC*, cutaneous squamous cell carcinoma; *HLA*, human leukocyte antigen; *SUNTRAC*, Skin and Ultraviolet Neoplasia Transplant Risk Assessment Calculator.

∗ High risk HLA included HLA DPB1∗18, HLA DPB1∗1801, HLA DRB1∗0103, HLA DPB1∗16, HLA DPB1∗1601, HLA C∗01, HLA C∗0102, HLA A∗2501, HLA B∗27, HLA B∗1501, HLA C∗0702, HLA B∗07.

Because the population was exclusively self-reported as white, there were no patients in the low-risk SUNTRAC group (Table I). The SUNTRAC-only model was consistent with published rates for skin cancer overall (C-statistic: 0.746, adjusted: 0.746), squamous cell carcinoma (C-statistic: 0.774, adjusted: 0.765), and basal cell carcinoma (C-statistic: 0.764, adjusted: 0.738) (Table II). Inclusion of HLA type did not have a significant impact for any skin cancer type (*P* > .05 for each).

**Table II:** Optimism-corrected concordance statistics for models including SUNTRAC and SUNTRAC plus HLA types

Model	Raw c-statistic	Optimism adjusted c-statistic (95% CI)	*P* value vs SUNTRAC only model
Skin cancer overall			
SUNTRAC only	0.746	0.746 (0.612-0.880)	N/A
SUNTRAC + HLA	0.756	0.753 (0.600-0.906)	.35
cSCC + cSCC-IS			
SUNTRAC only	0.774	0.765 (0.498-1)	N/A
SUNTRAC + HLA	0.771	0.755 (0.452-1)	.22
BCC			
SUNTRAC only	0.764	0.738 (0.348-1)	N/A
SUNTRAC + HLA	0.754	0.717 (0.314-1)	.32

*BCC*, Basal cell carcinoma; *cSCC*, cutaneous squamous cell carcinoma; *HLA*, human leukocyte antigen; *SUNTRAC*, Skin and Ultraviolet Neoplasia Transplant Risk Assessment Calculator.

Our retrospective analysis of 1499 white SOTRs showed no benefit from adding the HLA type to the SUNTRAC model. Our study further validated SUNTRAC’s ability to stratify cSCC risk and suggests that SUNTRAC’s capacity to predict skin cancer risk extends to basal cell carcinoma as well. All patients undergoing transplant will have HLA typing prior to transplant. The addition of HLA seemed promising from prior studies, although we did not observe any statistical difference with its inclusion.^3^

Our study acknowledges several limitations including the focus on a white SOTR population, missing data, and low power. Limiting to those with European genetic ancestry instead likely increased our power to detect differences in this more homogeneous population. There were less than or equal to 10 patients each with melanoma, melanoma in situ, and Merkel cell carcinoma. No analyses were attempted for these types due to very low power. HLA mismatch has been associated with cSCC risk in SOTR.^5^ Our de-identified study neither captured nor could link to donor HLA type, HLA mismatch, or percent panel-reactive antibodies for inclusion. Despite these limitations, our study demonstrates the robustness of the concise SUNTRAC model and suggests that it remains a valuable tool for clinicians, particularly when resources for additional testing are limited or when rapid risk assessment is required.

## Conflicts of interest

None disclosed.
